# Self-perception and perceived parental perception in adolescent girls with anorexia nervosa

**DOI:** 10.3389/fpsyg.2023.1301927

**Published:** 2024-01-19

**Authors:** Mariana Sokolov, Rachel Levy-Schiff, Adi Enoch-Levy, Daniel Stein

**Affiliations:** ^1^Safra Children’s Hospital, Chaim Sheba Medical Center, Ramat Gan, Israel; ^2^Department of Psychology, Bar Ilan University, Ramat Gan, Israel; ^3^Faculty of Medicine, Tel Aviv University, Tel Aviv, Israel

**Keywords:** anorexia nervosa, anxiety, depression, eating disorder, outcome, parents, social behavior, self-perception

## Abstract

Negative self-perception is associated with poor outcomes in adults with anorexia nervosa (AN). Our study aimed to assess the association between the self-perception of female adolescents with AN and how these adolescents perceive the attitudes of their parents toward them on the severity and short-term outcome of their illness. For this purpose, we assessed 30 adolescent girls hospitalized with AN and 30 female controls. Self-perception and perceived parental attitudes were assessed using the Structural Analysis of Social Behavior (SASB), according to which self-perception is formed via close relations with significant others in early life. Patients with AN responded to the SASB and to questionnaires assessing eating disorder (ED) symptomatology and emotional distress at both admission and discharge. Controls were similarly assessed once. We found that patients with AN showed a more negative self-perception than controls. Negative self-perception was associated with negative perceptions of the mothers’ attitudes toward the girls. There was no between-group difference in the perceived perception of the fathers’ attitude to the girls. Self-perception and perceived parental attitudes were associated with the severity of ED symptoms and emotional distress. Finally, an improvement was found in self-perception and perceived maternal attitudes toward the girl from admission to discharge, alongside a decrease in the severity of ED symptoms and emotional distress. Self-perception at admission was associated with ED pathology and emotional distress at discharge. These findings suggest that self-perception and perceived parental attitudes toward the adolescent with AN may be associated with the severity of the illness and its short-term outcome.

## Introduction

Eating disorders (EDs) are common in the modern world, with their frequency increasing in recent decades (Hoek, 2018). Anorexia nervosa (AN), usually starting during adolescence, is considered the most severe of all EDs in terms of earlier mortality and worse medical and psychological outcomes (Stein et al., 2009; Hoek, 2018).

The predisposition to the development of AN, currently considered a brain disorder rather than a psycho-socio-cultural disorder, is complex and multifactorial, involving primarily genetic and neurobiological factors (Hill et al., 2016). Nonetheless, many psychological correlations may be associated in this context with the development and maintenance of AN (Culbert et al., 2015). Among these correlations, negative self-perception has been identified as a risk factor for AN in adults, associated with a poor outcome (Ghaderi and Scott, 2001; Cervera et al., 2003). Compared with controls, patients with AN are characterized by a host of negative self-perceptions, including a highly self-critical attitude, perfectionistic self-demands, and negative bodily related attitudes (Ghaderi and Scott, 2001; Sassaroli et al., 2008).

According to several developmental theories (Winnicott, 1965; Mahler et al., 1975; Stern, 1985; Bowlby, 1988), self-perception may become organized via the internalization of early interpersonal relationships. Positive experiences with significant figures in childhood are of considerable importance in the attainment of positive self-perception and healthy development. Current research supports the idea that parental behavior perceived as positive (in terms of warmth) during childhood is linked to a positive self-image, whereas parental behavior perceived as negative (in the sense of absence of warmth) is linked to emotional difficulties, such as anxiety, depression, and juvenile delinquency (Wenar and Kerig, 2000; Hudson and Rapee, 2001; Muris et al., 2003).

Second, autonomy and separateness from the parents may also appear to be of importance. Adequate parental supervision has been linked to healthy development and better adjustment in adolescents, whereas excessive control and/or overprotective parenting may be linked to a range of emotional difficulties (Muris et al., 2003) and violent behavior (Aluja et al., 2005).

### The structural analysis of social behavior (SASB)

**Figure 1:**
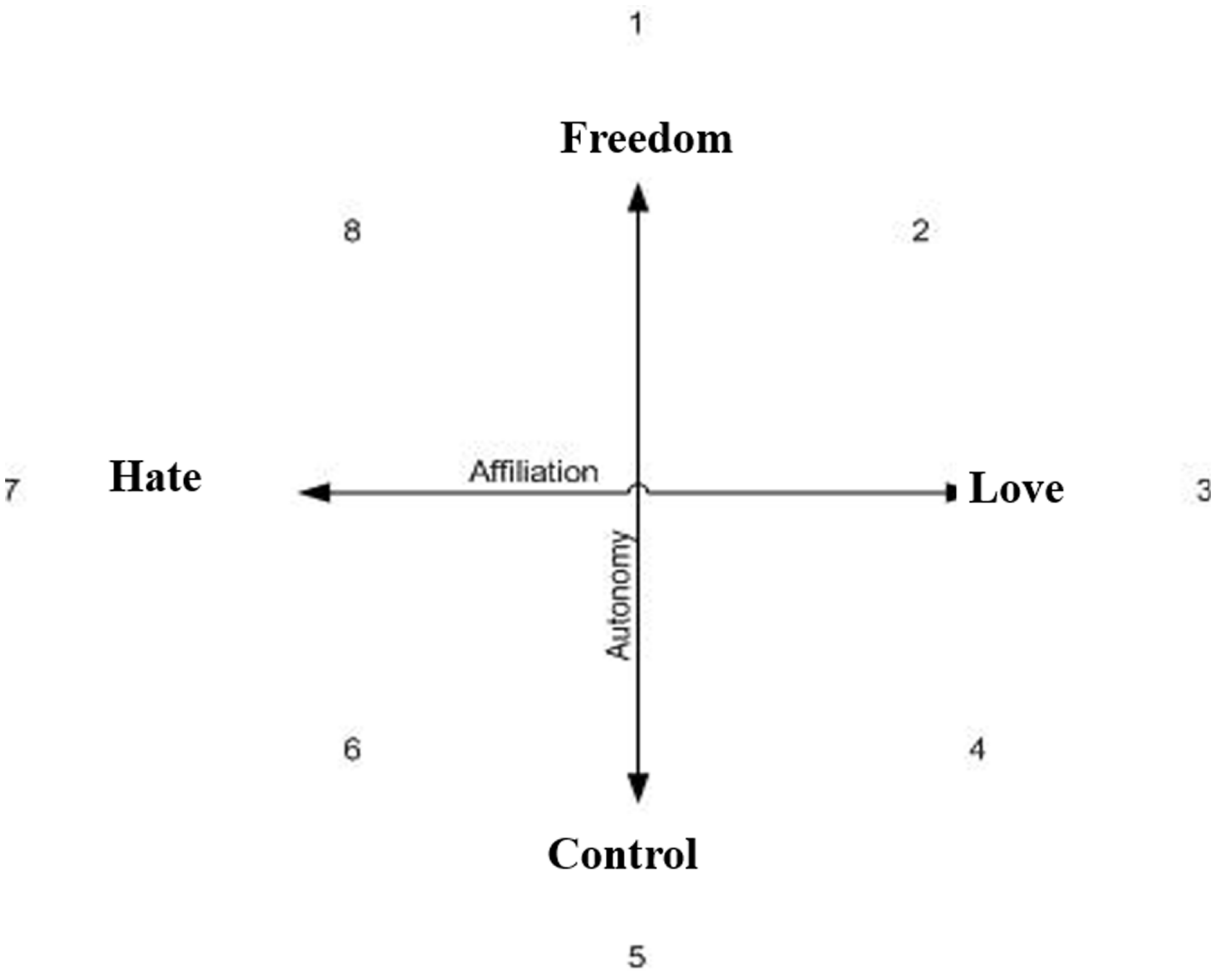
The structural analysis of social behavior (SASB) model.

Structural analysis of social behavior (SASB), developed by Benjamin (1974), is a psychological model of interpersonal relationships and intrapsychic representations. The model includes two axes referring to the dimensions of warmth and autonomy. The horizontal axis represents warmth (from love to hate), and the vertical axis represents autonomy (from freedom to control). The combination of both axes creates eight points of reference (see Figure 1) for object representations that contribute during the person’s development to his or her self-representations (Benjamin, 2000).

Studies have found that positive and stable self-perception in young adults (Benjamin, 1993) is linked to a positive perception of parental behaviors toward them in childhood in terms of warmth and autonomy. Adolescents perceive their relationships with their parents as warm and display fewer symptoms of depression and fewer behavioral problems (Benjamin, 2000). In contrast, adolescents with borderline personality attributes and antisocial tendencies, as well as with bulimia nervosa (BN), may show negative self-perception and negative perception of their parent’s behavior toward them (Armelius and Granberg, 2000). In particular, the perception of the parents as attacking may lead to the development of extreme negative self-perception (Ybrandt and Armelius, 2010).

### The SASB model in AN

Studies using the SASB model to examine self-perception in adult patients with EDs have found that these patients present with extreme negative self-perceptions characterized by excessive control, blame and criticism, and high levels of self-hate (Bjorck et al., 2003). These negative self-representations appear to be stable over time and difficult to change, even after several years of therapy (Birgegard et al., 2009).

A few studies examined self-perception in adolescents with EDs using the SASB. Humphrey (1989) found that adolescent girls with EDs were significantly more self-destructive than controls, showing less self-acceptance and self-care, and greater painful rejection and self-neglect than controls. Mantilla and Birgegard (2015), comparing the self-perception of patients with EDs aged 16–18 and 19–25, found stronger correlations between self-perception and ED symptoms in younger patients, with a weakening of these associations over time. Another study (Gezelius et al., 2016), examining the change in self-perception of adolescent patients with EDs following outpatient treatment, indicated, in contrast to the findings in adult patients, that young patients may show improved self-perception after treatment.

In light of these findings, we sought to conduct a longitudinal study in adolescents with AN, assessing self-perception from admission to inpatient treatment to discharge. In comparison to previous studies, we assessed not only the adolescents’ perception of themselves but also how they regard their parents’ perception of them. This was done to find out whether the way adolescents perceive their parents’ attitudes toward them would be associated with their own self-perception and, in turn, with their ED-related symptomatology and emotional distress. Our hypotheses were as follows:

1. Adolescent patients with AN would show more negative self-perception than control participants.
2. Adolescent patients with AN would perceive their parents’ attitudes to them as more negative than adolescent controls.
3. Self-perception would be associated with the way adolescents perceive their parents’ attitudes to them.
4. Self-perception and perceived parental attitudes would be associated with the severity of ED-related symptomatology and emotional distress at both admission and discharge.
5. The self-perception of adolescents with AN, as well as their perceived parental attitudes toward them, would improve following inpatient treatment, alongside an improvement in body mass index (BMI), ED-related pathology, and emotional distress.

## Materials and methods

### Participants

A total of 60 adolescent girls participated in this study. Of these, 30 were girls with AN who were hospitalized in the specialized department for the treatment of EDs in children and adolescents in the Safra Children’s Hospital, Sheba Medical Center, Tel Hashomer, Israel, between 2016 and 2020.

Inclusion criteria were adolescent girls, aged between 14 and 19 years, having a good understanding of the Hebrew language, and consent of the patients and their parents to participate in the study. Exclusion criteria were lifetime or current schizophrenic spectrum disorder, bipolar disorder, substance use disorder, organic brain disorder, developmental delay, any medical illness potentially affecting appetite or weight (e.g., diabetes mellitus or thyroid disorders), and chronic use of medications (except for psychotropic medications), defined as use of medications for no less than 4 consecutive weeks.

A DSM-4-TR (1997) diagnosis of AN was achieved using a semi-structured interview based on the principles of the Structured Clinical Interview for DSM-IV Axis I Disorder, Patient Edition, version 2.0; SCID-I/P version 2.0 (First et al., 1995). Some of the girls were actually diagnosed with atypical AN (eating disorder not otherwise specified-AN type). For the purpose of this study, case files were reviewed by the department’s child and adolescent psychiatrists, and the diagnoses were revised according to the DSM-5 (2013) criteria for AN. All patients with AN or atypical AN according to the DSM-4-TR (1997) were diagnosed with these diagnoses according to the DSM-5 (2013). It is of note that in both the DSM-TR and DSM-5, significantly low weight is defined as a weight that is less than minimally normal, or, for children and adolescents, less than that minimally expected, without requiring numerical criteria.

Control participants were a convenience sample of high school female students matched by residential area and age to the inpatients with AN. The controls were recruited by the research team. They were required to have a minimum BMI of 18.5 kg/m^2^.

### Instruments

The following self-rating questionnaires were used in the study.

*Eating Disorder Examination Questionnaire (EDE-Q;* Fairburn and Beglin, 1994*)*: The EDE-Q is a 36-item scale assessing restricting and binge–purge pathologies. Higher scores indicate greater pathology. The EDE-Q has been translated into Hebrew and validated in previous studies in Israel (Zohar et al., 2017; Gilon-Mann et al., 2018). The internal consistency of the total EDE-Q score in the present study is *α* = 0.721.

Depression was assessed using the 21-item *Beck depression inventory (BDI;* Beck et al.,1961*)* previously used in patients with EDs (Pollice et al., 1997), including in Israeli samples (Yackobovitch-Gavan et al., 2009). The internal consistency of the BDI in the present study was *α* =0.864.

*Depression Anxiety Stress Scale-21 (DASS-21;* Lovibond and Lovibond, 1995*)*: The Depression Anxiety Stress Scale-21 (DASS-21) is a 21-item scale assessing depression, anxiety, and stress. Higher scores indicate greater pathology. The DASS has been previously used in patients with EDs (Cardi et al., 2015; Stefanini et al., 2019), including in Israeli populations (Gilon-Mann et al., 2018). The Hebrew translation of the DASS has been validated in previous studies (Halpern et al., 2014). The internal consistency of the combined DASS score in the present study is *α* = 0.898.

In this study, we used the BDI instead of the DASS for the assessment of depression. In our opinion, although methodologically questionable, while the DASS assesses a combination of affective, cognitive, and somatic symptoms related to depression (anxiety and stress), the BDI adds further cognitive aspects that might be of greater importance in the assessment of the association of depression with self-perception.

*Structural Analysis of Social Behavior (SASB;* Benjamin, 1974*)*: Self and object representations have been measured with the SASB. This questionnaire refers to two dimensions represented by the following two axes: the horizontal axis is the affiliation aspect from love to hate, and the vertical axis is the autonomy aspect from control to freedom (see Figure 1). The two axes create the following eight clusters: emancipation, affirmation, love, protection, control, blame, hate, and neglect. These eight clusters represent the self and other perceptions toward the person. The SASB has three versions: long, medium, and short. We have decided to use the medium version, which has high validity but without a high burden to the respondent. In addition, the SASB can be used in references to different situations (me at my best/me at my worst, the other at his/her best/the other at his/her worst) and in different time periods (my parent’s attitude toward me at the age of 5–10; my parent’s attitude toward me currently).

In order not to overburden the participants, we chose to use two questionnaires of the SASB: A self-questionnaire that examines the participant’s attitude toward herself in the present, and a parent questionnaire (mother and father) that examines the perception of the participants regarding the attitudes of their parents toward them.

The self-questionnaire consists of 16 questions, two questions for each of the eight clusters. The mother/father questionnaire consists of 32 questions, four questions for each of the eight clusters. The participants are asked to rate their degree of agreement/disagreement with what is noted in the items on a scale from 0 to 100 (divided into 10 subscales, i.e., 0–9 and 10–19), where a score of less than 50 expresses disagreement (not true). Scoring above 50 expresses agreement (true).

The SASB has been previously used in patients with EDs (see Humphrey, 1989; Bjorck et al., 2003; Birgegard et al., 2009; Mantilla and Birgegard, 2015).

The SASB was translated into Hebrew by English-speaking translators knowledgeable in the field of EDs. It was later checked by two other English-speaking professionals, knowledgeable in the field of EDs. The internal consistency of the SASB in the present study was *α* = 0.906, and the test–retest reliability was *r* = 0.900.

### Procedure

All participants included in the study, and their parents in the case of minors under the age of 18, gave their written informed consent to participate in the study after receiving an explanation of the study goals and methodology. The study was described to both patients and controls as assessing the association of different psychological characteristics with different aspects of a person’s mental health. The study was approved by the internal review board (Helsinki Board) of the Sheba Medical Center, Tel Hashomer, Israel (Protocol No. 2755; 28 January 2016).

Patients with AN were interviewed at admission with the semi-structured interview based on the principles of the SCID-I/P version 2.0 by experienced child and adolescent psychiatrists. Diagnoses were confirmed in clinical team meetings of the department. Only patients for whom there was a unanimous agreement about their AN diagnosis were included in the study. Controls were excluded if they ever had a severe medical or emotional illness, except for intercurrent illness (asked with open-ended yes/no questions).

The principal researcher (MS), an experienced psychologist trained in the use of the SASB, administered this questionnaire to the participants. Other research assistants distributed the other questionnaires. All questionnaires were distributed in the morning hours in a random order.

The patient’s weight and height were taken during the morning hours by trained staff members after a night’s fasting according to standardized procedures (Tanner, 1994). The weight and height of the controls were self-reported. BMI was calculated as weight divided by height squared (Bray, 1992). Patients were assessed at both admission and discharge. Controls were assessed once.

Demographic and clinical variables, including age, BMI, and duration of inpatient treatment, were recorded using a demographic questionnaire and from the patients’ medical records.

### Statistical analysis

*T*-tests for independent variables were used for the comparison of baseline age and BMI of the research and control participants. *T*-tests for dependent variables were used for the comparison of admission and discharge age and BMI in patients with AN. Statistical comparisons were conducted using BMI raw data, rather than BMI percentiles, to optimize the statistical power of the calculations due to the larger sensitivity of the raw data. In addition, we used BMI and not BMI percentiles because we were dealing with adolescents, rather than with children (as noted in the inclusion criteria, girls had to be a minimum of 14 years old to be included in the study).

Part one of the study is a comparison between the research and control groups in the SASB variables (self, mother, and father perception), distress variables (depression, anxiety, and stress), and ED variables. Due to the non-normal distribution of some of the SASB variables, we used the Wilcoxon test for the comparison. A repeated measure multivariate analysis of variance (MANOVA) was performed to compare the ED and distress scales of AN patients and controls. A univariate analysis of variance (ANOVA) was used for the comparison of individual variables.

Part two is a comparison within the research group at admission and discharge from inpatient treatment of the SASB variables (self, mother, and father perception), distress variables (depression, anxiety, and stress), and ED variables. Due to the non-normal distribution of some of the SASB variables, we used a non-parametric Wilcoxon test for the comparison. A repeated measure MANOVA was performed to compare the ED and distress scales at admission and discharge. ANOVA was used for the comparison of individual variables.

Part three examines the relationships between the variables of self-perception, parental perception of the participant, and distress and ED measures. A Pearson correlation coefficient was used to examine the relationships among the variables.

## Results

### Demographic and clinical data

At admission, the mean age of the girls with AN was 15.71 (SD = 1.31) and the mean age of the girls in the control group was 16.41 (SD = 1.50). The difference in age between the two groups was not significant (*t* = −1.92; *p* = 0.06).

The mean age of patients with AN at discharge from inpatient treatment was 16.26 (SD = 1.37). The difference in age between admission and discharge was not significant (*t* = −1.59; *p* = 0.12). The mean duration of hospitalization was 207 days (SD = 80).

The mean baseline BMI of patients with AN [17.8 (2.06)] was significantly lower than that of the control participants [20.85 (1.60); *t* = −6.40; *p* = 0.001]. The difference between the admission [17.8 (2.06)] and discharge [20.45 (2.4)] BMI of the patients with AN was significant (*t* = −4.54; *t* = 0.001).

As noted earlier, some patients with AN did not have a low BMI when admitted to our inpatient ED department and were likely diagnosed with atypical AN. In this department, patients with AN are hospitalized not only because of low weight but also because of other reasons, including very rapid weight reduction, severe physiological decompensation, severe suicidality or comorbid psychiatric disturbance, and the inability of the family to maintain ambulatory treatment or failure of ambulatory treatment. Nonetheless, all patients had evidence of severe weight reduction at some period during their illness leading to a BMI of 15 kg/m^2^ or less. In the present study, six girls with AN had a BMI of 16 kg/m^2^ or less. No difference was found in the score of the psychometric scales in patients with AN with admission BMI being above (*n* = 18) or below (*n* = 12) 17 kg/m^2^, enabling us to relate to all research patients as one group of patients with AN.

### Part one—comparison of patients with AN and controls at admission

The between-group differences in self-perception (SASB) are summarized in Table 1. Patients with AN were characterized by a negative self-perception, compared with the controls, on 7/8 SASB scales, except for SASB self-neglect. Thus, they granted themselves less freedom and affirmation, loved and protected themselves less, and exhibited excessive self-control and a tendency to self-blame and self-attack.

**Table 1:** Differences between patients with AN and controls in baseline SASB self-perception.

Variable (SASB)	AN group *N* = 30	Control group *N* = 30	Z
	Mean rank	Mean rank	
Self-emancipation	23	34	2.56*
Self-affirmation	16	40	5.36***
Self-love	17	39	4.96***
Self-protection	21	36	3.41**
Self-control	36	23	3.03**
Self-blame	41	19	5.00***
Self-attack	37	22	3.53***
Self-neglect	25	33	1.90^NS^

AN, anorexia nervosa; SASB, Structural Analysis of Social Behavior; *p < 0.05; **p < 0.01; ***p < 0.0001; NS, not significant.

The between-group differences in the girls’ perceived attitudes of the mothers toward them are summarized in Table 2. Girls with AN perceived their mothers as granting them less freedom, blaming them more, and responding to them more angrily compared with the control girls.

**Table 2:** Between-group differences in the girls’ perception of the mothers’ attitudes toward them (only significant findings are presented).

Variable (SASB)	AN group *N* = 30	Control group *N* = 30	Z
	Mean rank	Mean rank	
Mother grants freedom	24	33	2.02*
Mother blames	34	25	2.09*
Mother is sulky (angry)	36	23	2.96*

N, anorexia nervosa; SASB, Structural Analysis of Social Behavior; *p < 05.

Only one difference was found in the SASB-perceived attitude of the father toward the girl, in that the fathers of the girls with AN were perceived by them as responding to them more angrily than control fathers [mean rank 34 vs. 24, respectively (*Z* = 2.30; *p* < 0.05)].

Significant differences were found between patients with AN and controls in the EDE-Q [*F* (5,55) = 15.71, *p* < 0.001, Eta^2^ = 0.5] and the combination of the BDI and DASS-21 anxiety and stress [*F* (3,57) = 33.48, *p* < 0.001, Eta^2^ = 0.64]. According to Table 3, patients with AN scored higher than controls on all measures of EDE-Q and DASS-21, as well as on the BDI.

**Table 3:** Between-group differences in baseline ED and distress variables.

Variable	AN (*n* = 30)	Controls (Variable = 30)	*F* (1,59)	Eta^2^
EDE-Q Restrictive eating	4.59 (1.33)	1.57 (1.64)	61.69***	0.51
EDE-Q Worries about food	2.61 (2.07)	0.46 (0.69)	29.70***	0.33
EDE-Q Worries about appearance	2.52 (1.36)	0.80 (0.64)	40.02***	0.40
EDE-Q Worries about weight	1.44 (1.17)	0.27 (0.37)	28.19***	0.32
EDE-Q Total	2.80 (1.00)	0.77 (0.74)	79.71***	0.57
BDI	33 (13)	8 (8)	76.79***	0.57
DASS-21 Anxiety	8.0 (5.0)	2.9 (3.5)	21***	0.27
DASS-21 Stress	13.0 (4.7)	4.7 (4.0)	53***	0.48

AN, anorexia nervosa; ED-eating disorder; EDE-Q, Eating Disorders Examination Questionnaire; BDI, Beck Depression Inventory; DASS-21, Depression, Anxiety, and Sress-21 Scale; ***p < 0.001.

### Part two—comparison of patients with AN at admission and discharge

Table 4 summarizes the differences between the admission and discharge of SASB self-perception. Self-affirmation and self-love increased at discharge, while self-control and self-blame were lower at discharge vs. admission.

**Table 4:** SASB self-perception of patients with AN at admission and discharge (only significant findings are presented).

Variable (SASB)	Admission (mean rank)	Discharge (mean rank)	*Z*
Self-affirmation	8	16	1.70*
Self-love	4	13	1.80*
Self-control	14	5	1.65*
Self-blame	15	8	2.17*

SASB, Structural Analysis of Social Behavior; AN, anorexia nervosa; *p < 0.05.

The differences in the SASB-perceived attitudes of the mothers toward the patients are presented in Table 5. Mothers were perceived by the patients as less blaming, less attacking, and less angry following inpatient treatment.

**Table 5:** SASB-perceived perception of the mother’s attitude toward the patient with AN at admission and discharge (only significant findings are presented).

Variable (SASB)	Admission (mean rank)	Discharge (mean rank)	Z
Mother blaming	9	3	1.92*
Mother attacking	7	1	2.32***
Mother sulky (angry)	16	6	2.57***

SASB, Structural Analysis of Social Behavior; AN, anorexia nervosa; *p < 0.05; ***p < 0.0001.

No significant differences were found in the SASB-perceived attitudes of the fathers toward the patients between admission and discharge.

A significant difference was found in the ED-related and distress variables between admission and discharge [*F* (3,23) = 4.30, *p* < 0.05, Eta^2^ = 0.36]. Table 6 summarizes the individual differences between the two conditions, showing a significant improvement in all parameters from admission to discharge.

**Table 6:** ED and distress scales of 30 patients with AN at admission and discharge.

Variable	Admission mean (SD)	Discharge mean (SD)	*F* (1,29)	Eta^2^
EDE-Q Restrictive eating	4.50 (1.35)	2.41 (1.73)	34.08***	0.57
EDE-Q Worries about food	2.58 (2.17)	1.22 (1.40)	9.40**	0.27
EDE-Q Worries about appearance	2.57 (1.42)	1.51 (0.76)	16.04***	0.38
EDE-Q Worries about weight	1.48 (1.22)	0.68 (0.52)	12.31**	0.32
EDE-Q Total	2.79 (1.05)	1.46 (0.93)	43.95***	0.69
BDI	33.07 (14.18)	25.11 (17.93)	9.789**	0.28
DASS-21 Anxiety	8.61 (5.64)	6.53 (5.58)	4.903*	0.16
DASS-21 Stress	13.61 (4.61)	11.15 (4.99)	5.348*	0.18

ED, eating disorder; AN, anorexia nervosa; EDE-Q, Eating Disorders Examination Questionnaire; BDI, Beck Depression Inventory; DASS-21, Depression, Anxiety, and Stress-21 Scale; *p < 0.05; **p < 0.01; ***p < 0.001.

### Part three—the relationship between SASB self-perception, SASB-perceived parental attitudes, and ED and distress measures

Owing to reasons of brevity, we describe the correlations between SASB self-perception, SASB-perceived parental perception, and ED and distress measures, in general, except for a more detailed description of the association of baseline SASB self-perception with ED pathology and distress at discharge. Accordingly, significant correlations were found between (1) baseline SASB self-perception (six scales) with baseline SASB-perceived maternal attitudes to the girl (seven scales), *r* = 0.26–0.46, *p* = 0.05–001; (2) baseline SASB self-perception (seven scales) with baseline SASB-perceived paternal attitudes to the girl (seven scales), *r* = 0.26–0.46, *p* = 0.05–001; (3) baseline SASB self-perception (six scales) with baseline ED pathology (EDE-Q restrictive eating, preoccupation with food, preoccupation with appearance, and EDE-Q total score) and emotional distress (DASS-21 anxiety and stress, BDI): *r* = 0.39–0.73, *p* = 0.05–0.001; (4) baseline SASB-perceived maternal attitudes (four scales) with baseline ED pathology (EDE-Q preoccupation with appearance, EDE-Q total score) and emotional distress (DASS-21 anxiety, BDI): *r* = 0.39–0.48, *p* = 0.05.

Finally, Table 7 describes the correlations between admission SASB self-perception and the ED and distress measures at discharge. The findings show that more negative relationships with oneself at admission (less self-affirmation, self-love, and self-protection, and more self-control, self-blame, and self-neglect) are associated at discharge with greater ED pathology, elevated DASS-21 stress and anxiety, and particularly elevated depression as assessed with the BDI.

**Table 7:** Correlations among SASB self-perception of patients with AN at admission and the ED and emotional distress measures at discharge.

ED and distress measures at discharge	Baseline SASB self-emancipation	Baseline SASB self-affirmation	Baseline SASB self-love	Baseline SASB self-protection	Baseline SASB self-control	Baseline SASB self-blame	Baseline SASB self-hate	Baseline SASB self-neglect
DASS-21 stress	−0.26^NS^	−0.09^NS^	−0.12 ^NS^	−0.001^NS^	0.43*	0.19^NS^	0.12^NS^	0.31^NS^
DASS-21 anxiety	−0.21^NS^	−0.15^NS^	−0.16^NS^	−0.008^NS^	**0.41** ^*^	0.11^NS^	0.08^NS^	0.24^NS^
BDI	−0.19^NS^	−0.52**	−0.79***	−0.58**	0.12^NS^	0.15^NS^	0.47*	0.39*
EDE-Restrictive eating	−0.23^NS^	−0.09^NS^	−0.06^NS^	−0.12^NS^	0.37*	0.12^NS^	0.04^NS^	0.18^NS^
EDE-Q Preoccupation with appearance	−0.29^NS^	−0.13^NS^	−0.21^NS^	−0.03^NS^	0.04^NS^	0.40*	0.03^NS^	0.27^NS^
EDE-Q Preoccupation with eating	−0.11^NS^	−0.16^NS^	−0.29^NS^	−0.28^NS^	0.19^NS^	0.33^NS^	0.29^NS^	0.30^NS^
EDE-Q Preoccupation with weight	−0.001^NS^	−0.14^NS^	−0.25^NS^	−0.29^NS^	0.09^NS^	0.28^NS^	0.17^NS^	0.12^NS^
EDE-Q Total score	−0.03^NS^	−0.09^NS^	−0.36^NS^	−0.28^NS^	−0.23^NS^	0.16^NS^	0.28^NS^	0.27^NS^

ED, eating disorder; AN, anorexia nervosa; SASB, Structural Analysis of Social Behavior; BDI, Beck Depression Inventory; DASS-21, Depression, Anxiety, and Stress Scale-21; EDE-Q, Eating Disorders Examination Questionnaire; *p < 0.05; **p < 0.01; ***p < 0.001; NS, not significant. Bold values mean significant findings.

## Discussion

### Between-group differences in SASB self-perception and perceived parental attitudes toward the girl

Developmental theories (Winnicott, 1965; Mahler et al., 1975; Stern, 1985; Bowlby, 1988) suggest that self-perception develops and becomes organized via early processes of internalization of interpersonal relationships. The general assumption underlying these theories is that the way significant others relate to a person in his or her early development may be connected with the individual’s future self-perception.

Our study was built in line with these theoretical assumptions, assessing self-perception, perceived parental attitudes to the girl, and the associations between them in patients with AN and control participants. The first hypothesis of the study, suggesting that adolescent girls with AN would show a more negative self-perception, was confirmed in that 7/8 SASB scales at admission were significantly more pathological in girls with AN compared with controls (only SASB self-neglect was not). The second hypothesis, suggesting that girls with AN would perceive their parents’ attitudes toward them as more negative in comparison with adolescent controls, was partially confirmed. Thus, girls with AN perceived the attitude of their mother as more negative on 3/8 SASB subscales compared to controls, relating to a sense of greater control over them and a more blaming and angrier attitude. In contrast, only one difference was found for the fathers, in that the fathers of girls with AN were perceived by the girls as responding to them more angrily. Third, as suggested in hypothesis 3, the girl’s self-perception was highly correlated with the way she perceived her mother’s and father’s attitudes toward her. Particularly, the lower self-emancipation (less freedom), self-affirmation, and self-love of girls with AN and their elevated self-control, self-blame, and self-attack were highly correlated with their mothers’ perceived elevated control and blaming.

Previous studies using the SASB have shown that adult patients with EDs have a highly negative perception of themselves, characterized by high levels of self-control, self-blame, and self-hate (Bjorck et al., 2003; Mantilla and Birgegard, 2015). The findings of the present study corroborate these findings in adults, showing a highly negative self-perception already in adolescent patients with AN. Thus, in comparison with healthy girls, girls with AN appear to grant themselves less freedom and affirmation, love and protect themselves less, and typically exhibit excessive self-control and a tendency to blame and attack themselves. In summary, the occurrence of the negative SASB self-perception profile in both adolescent and adult patients with an ED may suggest that negative self-perception might be found already relatively early in the course of AN, potentially continuing to later stages of the illness.

The findings of the present study suggest the likelihood of an association between the way the girls (both control and AN participants) see their parents’ attitudes toward them and their own perception of themselves. However, while control girls see the perception of both parents as more positive, perhaps associated with their own positive self-perception, the involvement of the father may be perceived by adolescent girls with AN as more positive and supportive (Keizer et al., 2019), amid the often more conflictual relationships with their mother (Ringler, 1996). These findings are of importance since, in one study, young patients with BN choosing not to involve their parents in their treatment have perceived their mothers as having a more blaming and negative attitude toward their illness (Perkins et al., 2005). While patients’ sex could have a role in the differences between fathers’ and mothers’ perceptions, it does not explain the differences found between the two female groups.

These findings should, however, be handled with caution. First, it is not clear from the cross-sectional correlational design of this study whether the relationships between self-perception and perceived parental attitudes toward the girl are unidirectional, bidirectional, or both. Thus, the group differences (patients with AN vs. healthy controls) in parental perceptions can putatively be related in part to the group differences in self-perception. In this context, the starved state that occurs during acute AN is often associated with increased low mood (Junne et al., 2016). A low mood may, in turn, be associated with increased self-criticism (Zhang et al., 2013; Gittins and Hunt, 2020), which could impact how individuals believe they are perceived by others (Joeng and Turner, 2015; Bonduelle et al., 2023). In addition, we are dealing with long-term memories of often treatment-resistant young girls in the midst of a very severe illness requiring hospitalization, often associated with a resultant familial crisis (Konstantellou et al., 2022), where the mother is likely the parent more involved in the treatment of the patient. Moreover, patients with AN may present with overgeneralized memory (Tenconi et al., 2021) and negative cognitive bias (Tenconi et al., 2023) that could compromise their memorizing abilities in the acute phases of their illness.

As expected (Junne et al., 2016), girls with AN have shown greater ED-related and comorbid anxiety and depressive symptomatology than control participants. Moreover, not only does the perceived relationship of the parents’ influence self-perception, but also as suggested in hypothesis 4, there appears to be a connection in our study between self-perception, the perceived relationship of the parents, and ED-related pathology (EDE-Q) and emotional distress (BDI, DASS-21). This finding adds the dimension of self-perception to studies showing an association between different core psychological characteristics, for example, self-esteem, compulsivity and impulsivity, and severity of ED and comorbid symptomatology (Fairburn et al., 1999; Howard et al., 2020). The novelty of our study, in this respect, relates to the associations found between the way the girl perceives her parents’ relationships with her, in addition to how she perceives herself, and the severity of her ED-related and comorbid emotional pathology.

### Comparison of the patient’s condition at the beginning and end of hospitalization

The findings of this part indicate an improvement from admission to discharge in 3/4 EDE-Q subscales, EDE-Q total, BDI, DASS-21 anxiety, and stress (Table 6) and BMI, alongside an improvement in SASB self-perception (Table 4), and perceived maternal attitudes toward the girl (Table 6). At the end of hospitalization, the girls grant themselves more freedom and affirmation, love and protect themselves more, and show less self-control, self-blame, and self-attack. Similarly, they experience their mothers as granting them more freedom and showing less blaming and angry attitudes. In other words, alongside the positive change in their relationship toward themselves following inpatient treatment, the girls also feel a change in similar dimensions in their mothers’ perception of them. The lack of change in the perceived parental attitudes may suggest lower involvement of the fathers in the treatment of their daughters during hospitalization. This finding is consistent with a study assessing SASB in parents of adolescents with AN, showing a change in mothers’ but not in fathers’ perceptions during an extensive outpatient program (Gezelius et al., 2016). Alternatively, the father’s attitudes have not been perceived by the girls with AN as more negative in comparison with control girls at the entrance to treatment. It is not surprising that these perceived attitudes have not changed at the end of treatment.

The improvement in emotional distress (depression, anxiety, and stress) and ED pathology following inpatient treatment, alongside a concomitant increase in BMI, is not surprising (Rothschild et al., 2009; Gezelius et al., 2016; Meule et al., 2021). This study enriches these data by showing a parallel improvement in both self-perception and perceived maternal attitudes. Similar to our findings, specific increases in SASB self-love and decreases in SASB self-blame, alongside an increase in BMI and symptomatic improvement following treatment, have also been noted elsewhere (Gezelius et al., 2016). The improvement in the SASB in adolescents with AN following a relatively brief intervention (approximately 7 months of hospitalization in our study) suggests that in contrast to adults, a negative self-perception is not yet stabilized in adolescents with AN, being likely more liable to improve following treatment (Gezelius et al., 2016). It further suggests that although self-perception can be considered an important risk factor for an ED, it is also a state-dependent variable, potentially changing alongside an improvement in ED and comorbid symptomatology. Finally, the significant correlations found between self-perception and perceived maternal attitudes may suggest that the improved self-perception at discharge, likely associated with an improvement in the physical and psychological state of the patients, can lead to a more benevolent perception of their mothers’ attitudes to them.

### The relationship of baseline SASB self-perception with ED and distress measures at discharge

In the present study, we found an association of both SASB self-perception axes—warmth and control—at admission, with the severity of ED-related and comorbid symptoms at discharge (see Table 7). These associations are particularly high for the BDI that includes additional cognitive dimensions of depression, in comparison with the more symptomatically oriented EDE-Q and DASS-21. This may suggest that treatment geared toward a change in perceptional aspects, whether related to the patient and/or to important figures in her life, would be associated more with the way the patient perceives her illness and recovery than directly influencing her actual symptoms. Again, these assumptions are only speculative, as the small number of the patients assessed does not enable us to suggest a model connecting self-perception, perceived parental perceptions, and the course of the illness.

Finally, given that a negative relationship with oneself at admission was associated with more ED-related pathology and greater emotional distress at admission, it would be relevant to assess whether the association between a negative relationship with oneself and greater pathology at discharge would still be significant after controlling for the impact of the baseline negative relationship to oneself. This result awaits further research.

### Limitations

As noted earlier, the findings of this study can be currently considered preliminary and speculative because of several limitations. First, our findings relied on the long-term memories of adolescent girls who were investigated during the acute stage of their illness, which likely would have influenced the way they perceived their parents, particularly their mothers. Second, the study was conducted with a relatively low number of participants. Although it currently enables the study of associations, it does not suggest a model for connecting self-perception, parental perceived attitudes toward the girls, ED and comorbid symptomology, and the course of the illness. Third, the controls represent convenience rather than a randomized controlled sample. They were not investigated in depth about the possibility of disturbed eating or another psychopathology. They were assessed only once, and their weight and height were self-reported, rather than measured. Fourth, the research group included both typical and atypical patients with AN. Nonetheless, the finding of significant differences between this group of patients and controls not only in ED and comorbid symptoms but also in core psychological characteristics such as self-perception suggests that it may be the illness *per se*, rather than the severe physiological condition at admission that is associated with negative self-perception. Fifth, we had not assessed for comorbidity in the AN group, although depression, anxiety, and stress have been measured via self-rating scales. Finally, as the AN group included only inpatients, our findings could not be generalized to patients with less severe illness.

Our study has nevertheless some important advantages. It is a hypothesis-generated prospective longitudinal study, and we have used adequate assessment tools. The novelty of our findings relies on the assessment of perceived parental attitude toward the girl in addition to self-perception, allowing us to speculate about the importance of the inter-relationships of these dimensions in the predisposition to and maintenance of AN.

## Conclusion

The findings of this research indicate that female adolescents with AN show more negative self-perception than control female adolescents and regard the perception of their mothers, but not their fathers, as more negative. Significant associations have been found between self-perception and perceived parental attitudes and between self-perception and perceived parental attitudes with the severity of ED-related symptomatology and emotional distress at both admission and discharge. Finally, the self-perception of adolescents with AN, as well as their perceived maternal (but not paternal) attitudes toward them, improved following inpatient treatment, alongside an improvement in BMI, ED-related pathology, and emotional distress. These findings highlight the importance of a multi-faceted treatment of AN as early as possible, not only to prevent the continuation of ED-related and comorbid symptoms but also to improve the perception of the girl herself and to intervene in the relationships of the ill girl with her parents.

### Recommendations for future research

Future prospective longitudinal research should include a larger number of ambulatory adolescents with different types of EDs, alongside a larger number of randomized controlled well-defined controls. This would enable an examination of causality, rather than only correlations between variables. Parents should also be included in the study to assess whether there would be a difference in the way the patients regard their parents’ perception of them and the actual parental perception of their children (both boys and girls). Such a prospect would enable us to examine whether self-perception and perceived and actual parental attitudes toward the girls would have a role in the course and outcome of an ED.

## Author’s note

The work was part of the requirements of PhD in clinical psychology, Department of Psychology, Bar Ilan University, Israel.

## Data availability statement

The raw data supporting the conclusions of this article will be made available by the authors, without undue reservation.

## Ethics statement

The study involving human participants was reviewed and approved by the Ethics Committee [Internal Review Board (Helsinki Board) of the Sheba Medical Center, Tel Hashomer, Israel (Protocol No. 2755; January 28th, 2016)]. Written informed consent to participate in this study was provided by all patient/participants and their legal guardian/next of kin in the case of minors under the age of 18. The studies were conducted in accordance with the local legislation and institutional requirements.

## Author contributions

MS: Conceptualization, Data curation, Formal analysis, Writing – original draft, Writing – review & editing. RL-S: Conceptualization, Writing – original draft, Writing – review & editing. AE-L: Investigation, Project administration, Writing – original draft. DS: Conceptualization, Data curation, Investigation, Writing – original draft, Writing – review & editing.
